# Development of cancer genetic services in the UK: A national consultation

**DOI:** 10.1186/s13073-015-0128-4

**Published:** 2015-02-02

**Authors:** Ingrid Slade, Daniel Riddell, Clare Turnbull, Helen Hanson, Nazneen Rahman, for the MCG programme

**Affiliations:** The Institute of Cancer Research, 15 Cotswold Road, Sutton, Surrey SM2 5NG UK; The Ethox Centre, Nuffield Department of Population Health, University of Oxford, Old Road Campus, Oxford, OX3 7LF UK; Centre for Personalised Medicine, Wellcome Trust Centre for Human Genetics, St Anne’s College, University of Oxford, Oxford, OX2 6HS UK; Royal Marsden NHS Foundation Trust, Downs Road, Sutton, Surrey SM2 5PT UK

## Abstract

**Background:**

Technological advances in DNA sequencing have made gene testing fast and affordable, but there are challenges to the translation of these improvements for patient benefit. The Mainstreaming Cancer Genetics (MCG) programme is exploiting advances in DNA sequencing to develop the infrastructure, processes and capabilities required for cancer gene testing to become routinely available to all those that can benefit.

**Methods:**

The MCG programme held a consultation day to discuss the development of cancer genetics with senior representation from all 24 UK cancer genetic centres. The current service landscape and capacity for expansion was assessed through structured questionnaires. Workshop discussion addressed the opportunities and challenges to increasing cancer gene testing in the National Health Service (NHS).

**Results:**

Services vary with respect to population served and models of service delivery, and with respect to methods and thresholds for determining risk and testing eligibility. Almost all centres want to offer more cancer gene testing (82%) and reported increasing demand for testing from non-genetic clinical colleagues (92%). Reported challenges to increasing testing include the complexity of interpreting the resulting genetic data (79%), the level of funding and complexity of commissioning (67%), the limited capacity of current processes and cross-disciplinary relationships (38%), and workforce education (29%).

**Conclusions:**

Priorities to address include the development and evaluation of models of increasing access to gene testing, the optimal process for interpretation of large-scale genetic data, implementation of appropriate commissioning and funding processes, and achieving national consistency. The UK cancer genetics community have high expertise and strong commitment to maximising scientific advances for improved patient benefit and should be pivotally involved in the implementation of increased cancer gene testing.

**Electronic supplementary material:**

The online version of this article (doi:10.1186/s13073-015-0128-4) contains supplementary material, which is available to authorized users.

## Background

Healthcare services need to utilise advances in genomic technology and knowledge for the benefit of patients. This was a key theme of the 2003 Genetics White Paper *Our Inheritance, Our Future* [1]. Since 2003 there has been rapid, revolutionary development of genomic technologies and extensive advances in knowledge of the impact of genomic variation on human disease. High-throughput DNA sequencing and analysis have been widely used in the research setting, but have not, thus far, been incorporated at any scale into clinical service provision in the National Health Service (NHS) [2,3]. The House of Lords Genomic Medicine Report highlighted this translational gap and began to define the challenges of integrating genomic advances within the NHS [4]. However, despite 10 years of discussions there remains a lack of clear operational direction regarding the implementation of increased gene testing within healthcare services.

In the UK, specialist genetic services, including cancer genetics, are organised regionally and delivered by consultant clinical geneticists and genetic counsellors [5]. Cancer genetics offers services to individuals and families with the goal of assisting treatment decisions in patients with cancer and facilitating early cancer detection and cancer prevention in their relatives. This includes cancer risk estimation, gene testing, assistance with decisions on treatment options, referral for cancer surveillance and discussion of cancer risk-reducing options. Mutations of over 100 genes are known to cause an increased risk of cancer and these underlie approximately 3% of cancer overall, though the contribution to individual cancers varies widely, with substantial contribution to some cancers such as childhood embryonal tumours, ovarian cancer and certain endocrine cancers [6]. Furthermore there is increasing evidence that identification of cancer predisposition gene mutations has an impact on improved diagnosis and management of cancer patients and their families [6].

A large proportion of the work of any cancer genetic service is the management of familial breast and ovarian cancer, and this clinical area exemplifies both the opportunities and challenges to increasing access to gene testing. Management of familial breast cancer is one of the few areas of clinical genetic practice with National Institute of Health and Care Excellence (NICE) Guidance [7]. Mutations in *BRCA1* and *BRCA2* underlie a proportion of breast and ovarian cancer and the most recent guidelines for familial breast cancer, updated in 2013, recommend testing individuals at ≥10% chance of having a mutation [7,8]. Studies have shown that approximately 15% of high-grade serous ovarian cancer is due to mutations in *BRCA1* or *BRCA2* [9,10]. Importantly, about half of the mutation-positive patients do not report a significant family history of cancer [9]. Although eligible for testing at the current risk threshold, rate of referral of ovarian cancer patients to clinical genetics is low, reported to be only 7% to 20% [9,10]. In addition to the inequity compared to familial breast cancer, important opportunities for improved management of ovarian cancer patients and cancer prevention in their relatives are being lost. The current model of specialised service delivery, with limited staff numbers, inflexible infrastructure and specialised commissioning, lacks the capacity to accommodate this unmet obligation, or to address increasing demand for cancer gene testing more generally.

The Wellcome Trust Mainstreaming Cancer Genetics (MCG) programme [11] is a cross-disciplinary, translational initiative that aims to develop the assays, informatics, clinical infrastructure, education and evaluation that will allow implementation of gene testing into routine clinical care of cancer patients and their relatives. The programme aims to provide scalable and transferable tools for the integration of gene testing into mainstream medical practice across the NHS [12]. The MCG programme hosted a consultation day in July 2013, to inform the development of its implementation models. The consultation included senior clinical representatives from all 24 cancer genetic services across the UK. The list of cancer genetic services represented is given in Additional file 1: Table S1.

## Methods

### Selection of consultation representatives

A list of all UK cancer genetic services was compiled using information available on the British Society for Genetic Medicine (BSGM) website [13]. A total of 24 UK cancer genetic services were identified. All the cancer genetic leads from these services were contacted and invited to attend the consultation event, or to nominate a senior representative of their service if they were personally unable to attend. All services invited agreed to attend. The attendees were sent two pre-consultation questionnaires to complete prior to the event. We encouraged the attendees to discuss and answer the questionnaires with their colleagues, so that the answers were representative of their service. All attendees at the consultation event participated in the workshop discussions. It was agreed that individual responses would be kept confidential to ensure representatives could respond freely.

### Pre-consultation questionnaires

Two structured questionnaires were sent by email to each attendee prior to the day. The Service questionnaire focused on the current landscape of cancer genetic services (Additional file 2: Table S2). The Implementation questionnaire addressed attitudes and capacity for evolution of services (Additional file 3: Table S3). The questions in the pre-consultation questionnaires were selected, after detailed discussions, by five members of the MCG programme team (IS, DR, CT, HH and NR) informed by their extensive experience of the field and the engagement activities of the MCG programme. Multiple choice and discrete answers from the questionnaires were collated directly; free text responses were independently curated by two MCG programme members (IS and DR) and then collated.

### Focused workshop discussion

The consultation day included three focused workshop discussions. Each workshop addressed a defined question: (1) How can we provide equitable gene testing to ovarian patients and their families? (2) How can we ensure variants are interpreted and managed appropriately? (3) How can we implement consistent cancer genetic care across UK? The topics were selected by five members of the MCG programme team (IS, DR, CT, HH and NR) after evaluation of the pre-consultation questionnaires to allow more focused, detailed discussions of the areas causing biggest concern. The consultation day attendees were divided into four groups, each with a facilitator (NR, IS, HH or CT) to discuss each question in turn. This was to ensure opportunities for all attendees to voice their opinions. The groups were organised such that the location and size of services was evenly distributed. After each workshop discussion the full group reconvened to feedback individual group viewpoints and then discuss the question together. The outcomes of the workshop discussions were documented separately (NR, IS, HH or CT) and reviewed and collated afterwards (IS and DR). Notes of the full consultation day discussions and pre-questionnaire data were documented and summarised separately (IS and DR) and then collated and discussed (IS, DR, CT, HH and NR).

### Consultation report

Prior to writing the manuscript a summary of the consultation outcomes was circulated to all the attendees and also presented at the British Society for Genetic Medicine conference, to ensure it was a fair and appropriate representation of the consultation discussions. The manuscript was also circulated to the attendees. It should be noted that this report is a summary of the consultation and therefore does not necessarily represent individual attendees’ or genetic centres’ viewpoints.

## Results and discussion

### Service and implementation questionnaires

Twenty-two responses were received for the service questionnaire (92%). Twenty-four responses were received for the implementation questionnaire (100%).

#### Overview of cancer genetic services

In the UK, cancer genetic centres provide a service for populations between 475,000 to 5.4 million people. Each centre works with between one and seven specialist oncology centres in providing their service. All services have a team of consultant cancer geneticists, specialist registrars in training and genetic counsellors. Individual centres differ with respect to the thresholds at which testing for a *BRCA1* or *BRCA2* mutation is offered and also in the methods used for determining risk. The risk thresholds were in the range of 10% to 20%. Many different methods for determining risk are currently used, including local or multi-regional protocols based on practical distillation of modelled data, for example, the Marsden Protocol 2 [14] to genetic evaluation of each patient by individual clinicians facilitated by tools such as the Manchester scoring system and/or computer-based algorithms such as BOADICEA [15].

#### Current capacity

Within their current service configuration 14% (3/22) of centres said they were unable to offer testing to additional people. Fifty-five percent (12/22) said they could accommodate moderate increase in testing; between 50 and 150 extra people per year. Twenty-seven percent (6/22) responded that their current service could accommodate testing >150 extra people per year. There was one non-response.

Individuals with high-grade serous ovarian cancer, with or without a family history of breast or ovarian cancer are at a greater than 10% risk of having a *BRCA1* or *BRCA2* mutation [9,10]. In response to this evidence the majority of UK cancer genetic centres (73%, 16/22) are planning to at least offer testing to ovarian cancer patients that are referred to their service. However, 27% (6/22) of centres reported not being able to do so, all stating insufficient funding and/or staff as the reason.

One of the models proposed to increase gene testing is for non-genetic specialist services to undertake testing in eligible patients within their area of clinical expertise [12]. Sixteen centres (73%, 16/22) reported that non-geneticists within their region are already directly ordering specific tests, by local agreement. The majority of this ‘mainstream’ delivery of cancer gene testing is for *RET* testing by endocrinologists (36%, 8/22). *BRCA1* and *BRCA2* testing by oncologists is occurring in three centres (14% - including The Royal Marsden Hospital through an implementation pilot of the MCG Programme - see [11] for more details).

#### Future service expansion

The great majority of the UK cancer genetic centres, 82% (18/22, two non-responses), would like to be able to offer more cancer gene testing. Moreover, 92% (22/24) think there is/will be increasing interest from non-genetic clinicians in their region for more cancer gene testing in their patients. All centres believe there is/will be increasing interest from patients and the public about cancer gene testing.

#### Concerns and barriers

The greatest concern expressed about increasing gene testing was the challenge of interpreting the increased number of variants that would inevitably be identified. Nineteen of the 24 centres (79%) highlighted this as a concern. The second most commonly reported concern was inadequate or mis-timed pre-test counselling of patients (38%, 9/24).

Funding, both the level funding and the complexity of commissioning was identified as the most significant barrier to increasing gene testing, highlighted by 16/24 (67%) centres. Other barriers included the limited capacity of current processes and the paucity of cross-disciplinary relationships (38%) and workforce education (29%).

### Focused workshop discussions

#### How can we provide equitable gene testing to ovarian cancer patients and their families?

All of the cancer genetic centre representatives were in full agreement that provision and access to gene testing for ovarian cancer patients needs to be improved. Many attendees proposed this could be achieved by integrating gene testing within the gynae-oncology services, with close liaison and support from genetics. The discussion highlighted significant challenges to this approach, most immediately education and funding. It was agreed that successful integration of cancer gene testing into oncology services would require consistent, clear, user-friendly guidelines to ensure high-quality, robust, equitable care pathways. The importance of ensuring the expertise of cancer genetic services was fully utilised in the development and implementation of increased testing for ovarian cancer patients across the UK was particularly highlighted.

#### How can we ensure variants are interpreted and managed appropriately?

Sequencing of a gene, by any method, will identify sequence variants, many of which will alter the amino acid sequence of the resulting protein. Thus clinical genetic services are familiar with the need to interpret genetic variants. However, there is little formal educational or professional support for clinicians in the interpretation and clinical management of variants, and decisions are based on individual ‘expert’ opinion and experience. Furthermore, for most cancer predisposition genes there are no validated functional assays or computational tools that can robustly predict the clinical impact of most variants [16]. The consultation group identified that the current specialist genetic workforce were not adequately trained or supported in the clinical interpretation of variants. Together with the absence of guidance for the systematic clinical interpretation of variants, this has resulted in inconsistencies in approaches to both test reporting and variant management in the UK.

Reliance on individual expert opinion is not scalable and its limitations will be magnified with increased cancer gene testing and the subsequent increased volume of data to be interpreted. The consultation group discussed the imperative for national consistency in reporting and interpretation of variants, which should include clear information about the clinical relevance and should be readily triaged into management recommendations. The growing momentum for mainstream (non-genetic) speciality consultants to order gene tests places further urgency on the development of robust, professionally agreed, national, standardised systems of reporting, to ensure variants are interpreted and managed consistently and appropriately.

#### How can we implement consistent cancer genetic care across the UK?

It was agreed that consistency of care should be a central tenet of the NHS cancer genetic services. It was recognised that there is an existing need for improved consistency of service delivery, methods for calculating test eligibility and risk thresholds, and patient and variant management. There should be uniformity within and between genetic centres and this should also extend to genetic care delivered outside the specialist genetic centres, as increased mainstreamed testing evolves. To promote and facilitate this there is a need for nationally agreed standardised guidelines that are evidence-based and simple to understand.

While the consultation group acknowledged that there will always be complex cases that fall outside of guidance, they agreed that protocols would be able to guide care for the majority of patients. Specific challenges identified that can impede delivery of consistent genetic care include the rapid evolution of the field, frequent guideline updates and insufficient funding available to enable successful and consistent implementation of guidelines into clinical practice nationally.

## Conclusions

With the rapidly expanding knowledge of how genetics can impact health and disease, there is a need for genetic medicine to be more widely available [17]. Moreover, improvements in the speed and affordability of DNA sequencing have opened up enormous opportunities, in numerous areas of medicine, to improve healthcare. It is generally acknowledged that there is insufficient capacity for specialist clinical geneticists to provide the genetic elements of clinical care for all patients in all clinical areas, either to meet current or future demand. It has been proposed that ‘mainstreaming’ of genetic services with incorporation of many aspects of genetic medicine into the care pathways of other medical specialities is the best solution [12,18,19]. However, movement towards this model has been slow.

Cancer genetics is one of the foremost areas where gene testing can impact healthcare. Cancer gene testing is becoming increasingly relevant to the healthcare needs of oncology patients impacting on diagnosis, management and prognosis as well as having potential to improve cancer risk information for their relatives. Consultation with the UK cancer genetic services reported here demonstrates the increasing demand from clinicians and patients across the UK for cancer gene testing. The key recommendations from the day are outlined below.

There was consensus that the current system of gene testing, which is largely restricted to patients seen by specialist cancer genetic services, does not have capacity to accommodate the increasing demand for cancer gene testing. There was broad, though not universal, support for mainstreaming of gene testing, that is, integration of testing into routine patient pathways in oncology in close liaison with genetic services. It was felt this would likely prove the optimal pathway for most cancer patients, however appropriate training of non-geneticists would be required and close liaison with genetics, who should continue see all patients found to have a mutation or unclassified variant and any complex cases, was felt essential.

Consistency of care should be a central tenet of both the cancer genetic services and any future mainstreamed gene testing within oncology. The most robust method for achieving this will be through nationally agreed evidence-based guidelines. It is essential that such guidelines are designed to easily allow iterative review and incorporation of up-to-date evidence in this fast-moving field of medicine.

The key challenges identified are summarised below. The greatest concern to those working within the cancer genetic services is in relation to the clinical interpretation and management of genetic variants. There is an existing urgent need for systematic guidance to aid the consistent clinical interpretation of variants. In the near future the volume of variants requiring interpretation will greatly increase as the data being generated across the healthcare sector are increasing rapidly through increased single gene, panel, exome and whole genome testing [12,20,21]. Accurate and reliable clinical interpretation of this large volume of data will be required for the full clinical utility of genomic medicine to be realised [20,22]. Automated, systematic, evidence-based variant interpretation guidelines/pipelines that can routinely address 90% to 95% of variants will be required for integration of genomic medicine with mainstream specialties. Such a system must also be dynamically responsive to new evidence [22,23]. The remaining 5% to 10% of more complex variants/cases will likely continue to require expert interpretation by specialists.

Sufficient funding and appropriate resource allocation are key challenges. Realising a healthcare system where genetic information is integrated in standard medical care is a complex task that will require sustained effort and resources within organisational structures receptive to change [17]. Commissioning is a key mechanism for delivering equitable high quality services that incorporate genetics and genomics across the UK. The system of specialist commissioning, in which clinical genetics is currently housed, seems at odds with a model of ‘mainstreaming’ for service delivery. This clear disjunction between ‘specialised’ commissioning and delivery of ‘mainstreamed’ services will ultimately demand a novel approach to resource allocation in order to incorporate a growing volume of gene testing within the healthcare service as a whole.

In the genetics white paper the government stated its ambition for a ‘National Health Service to incorporate genetics in every sector’, although how this is to be achieved remains unclear [1]. The implementation of genomic medicine within the NHS is an ambition that demands action and operational leadership beyond rhetoric. Building on the insights from the cancer genetic services consultation day the MCG programme is optimising and evaluating a ‘mainstream’ model of gene testing whereby the test in cancer patients is performed within cancer services, underpinned by the expert support of genetics. Additional priorities will be to develop systematic approaches for the clinical interpretation of large-scale genetic data that will result from adoption of genomic medicine.

### Key recommendations

1. Mainstreaming of cancer gene testing, with tests in cancer patients performed through the routine cancer patient pathway, is likely to be the optimal approach to deliver the required volume of tests.
2. Mainstreaming should be implemented in collaboration with genetics, who should continue to see any individuals found to have mutations, and any complex cases.
3. Nationally agreed, evidence-based, simple guidelines outlining eligibility for gene testing are required to ensure national consistency.
4. Consistent clinical interpretation of variants is required. Improved training and support for geneticists in clinical variant interpretation together with improved automated interpretation pipelines should be developed.
5. A sustainable model of resource allocation that promotes and supports this mainstreamed model of service delivery is required.

### Key challenges identified

1. Lack of capacity of cancer genetic services at a time of greatly increased demand for cancer gene testing from patients and clinicians.
2. Inconsistency of services and patient management, particularly with regard to testing eligibility, risk estimation and variant interpretation practices.
3. Education of non-geneticists to ensure appropriate information and support is provided to the patients they test.
4. Improved education of genetic services in clinical interpretation and management of genetic variants.
5. Sufficient and appropriately configured funding.

## Additional files

Additional file 1: Table S1. Cancer genetic services represented. *The attendee from the Oxford Regional Genetics Service completed the pre-consultation questionnaires but was unable to attend the consultation day. Additional file 2: Table S2. Service questionnaire. Additional file 3: Table S3. Implementation questionnaire.

## Abbreviations

BOADICEA: Breast and Ovarian Analysis of Disease Incidence and Carrier Estimation Algorithm
BRCA1/2: Breast cancer 1/2, early onset
DNA: Deoxyribonucleic acid
ICR: Institute of Cancer Research
MCG: Mainstreaming Cancer Genetics
NHS: National Health Service
NICE: National Institute of Health and Care Excellence
RET: Ret proto-oncogene
UK: United Kingdom
