# Supplementary material for: Development of cancer genetic services in the UK: A national consultation
Source: Genome Med. 2015 Feb 2;7(1):18. doi: 10.1186/s13073-015-0128-4 (PMC4341881; doi:10.1186/s13073-015-0128-4)
Supplement: Additional file 1: Table S1. — Cancer genetic services represented. *The attendee from the Oxford Regional Genetics Service completed the pre-consultation questionnaires but was unable to attend the consultation day. [file 13073_2015_128_MOESM1_ESM.doc]

**Supplementary Information**

**Additional file 1: Table S1. Cancer Genetic Services Represented**

| **Centre** |
| --- |
| Yorkshire Regional Genetics Service |
| Wessex Clinical Genetics Service |
| Leicestershire Genetics Centre |
| East of Scotland Genetic Service |
| North West Thames Genetics Service |
| Northern Genetics Service |
| Peninsula Regional Genetics Service |
| South West Thames Regional Genetics Service |
| Sheffield Regional Genetics Service |
| West of Scotland Regional Genetics Service |
| South Western Regional Genetics Service |
| Merseyside and Cheshire Clinical Genetics Service |
| The Royal Marsden Cancer Genetics Unit |
| Manchester Centre for Genomic Medicine |
| North of Scotland Genetic Service |
| Northern Ireland Regional Genetics Centre |
| West Midlands Regional Genetics Service |
| All Wales Medical Genetics Service |
| Nottingham Regional Genetics Service |
| SE Thames Regional Genetics Service |
| NE Thames Regional Genetics Service |
| South East Scotland Genetic Service |
| East Anglian Regional Genetics Service |
| Oxford Regional Genetics Service* |

*****The attendee from the Oxford Regional Genetics Service completed the pre-consultation questionnaires but was unable to attend the consultation day
