# Supplementary material for: Development of cancer genetic services in the UK: A national consultation
Source: Genome Med. 2015 Feb 2;7(1):18. doi: 10.1186/s13073-015-0128-4 (PMC4341881; doi:10.1186/s13073-015-0128-4)
Supplement: Additional file 2: Table S2. — Service questionnaire. [file 13073_2015_128_MOESM2_ESM.doc]

**Additional file 2: Table S**2. Service Questionnaire

| **Question number** | **Question** |
| --- | --- |
| 1 | What is your name? |
| 2 | What size population does your genetic service cover and how many cancer genetic consultations does your service do each year? |
| 3 | How is your cancer genetic service configured?  i.e. who sees the patients (consultants / SpRs / counsellors) and how does triage take place (through FHx clinics / telephone clinics, no triage etc)? |
| 4 | How many oncology centres do you work with? |
| 5 | Within your service are any cancer gene tests ordered by non-geneticists (e.g. RET tests by endocrinologists or BRCA tests by oncology)? |
| 6 | What threshold are you using for BRCA testing and how do you decide who to test for the BRCA gene mutations? Please email your protocol to mcg@icr.ac.uk if available. |
| 7 | The revised NICE guidelines recommend people with 10% risk of having a BRCA mutation should have access to testing. Will your service be able to seamlessly accommodate this? If no, what changes will be required? |
| 8 | 70% of ovarian cancer patients meet the 10% threshold irrespective of family history. Is your service offering, or planning to offer, testing to all ovarian cancer patients that meet a 10% threshold? If yes, how will this be accommodated? If no, why not? |
| 9 | How much increased gene testing could your service provide with your current systems? |
| 10 | Is your service doing any activities with respect to increasing cancer gene testing and/or mainstreaming? If yes, please expand |
