# Supplementary material for: Development of cancer genetic services in the UK: A national consultation
Source: Genome Med. 2015 Feb 2;7(1):18. doi: 10.1186/s13073-015-0128-4 (PMC4341881; doi:10.1186/s13073-015-0128-4)
Supplement: Additional file 3: Table S3. — Implementation questionnaire. [file 13073_2015_128_MOESM3_ESM.doc]

**Additional file 3: Table S**3. Implementation Questionnaire

| **Question number** | **Question** |
| --- | --- |
| 1 | What is your name? |
| 2 | Would you personally like to be able to offer more cancer gene testing? If yes, which tests and/or to which people? |
| 3 | Do you think there is/will be increasing interest from non-genetic clinicians (e.g. oncologists) in your region to have more cancer gene testing in their patients? If yes, which tests and/or in which patients? |
| 4 | Do you think there is/will be increasing interest from patients and/or the public to have cancer gene testing? |
| 5 | Do you think it would be helpful to be able to test lots of cancer genes at once, for example the BRCA and Lynch genes in an ovarian cancer family? |
| 6 | Do you think if you tested a cancer gene panel and found a mutation in a gene not normally associated with the patient’s cancer it would constitute an ‘incidental finding’?  If yes, do you think it appropriate to tell the patient? |
| 7 | Do you feel confident about molecular genetic results, particularly interpreting the likely clinical consequences of ‘variants of unknown significance’? |
| 8 | What are your greatest areas of concern with respect to increasing cancer gene testing? |
| 9 | What do you think are the greatest barriers to increasing cancer gene testing? |
| 10 | What outcomes are you hoping for from the MCG Consultation Day on 1st July? |
